# The association between the multiple birth and breast cancer incidence: an update of a systematic review and meta-analysis from 1983 to 2022

**DOI:** 10.1186/s13690-023-01089-0

**Published:** 2023-04-28

**Authors:** Pedram Veisi, Maziar Nikouei, Mojtaba Cheraghi, Sholeh Shahgheibi, Yousef Moradi

**Affiliations:** 1 Student Research Committee, Kurdistan University of Medical Sciences, Sanandaj, Iran; 2 Department of Obstetrics and Gynecology, School of Medicine, Besat Hospital, Kurdistan University of Medical Sciences, Sanandaj, Iran; 3 Social Determinants of Health Research Center, Research Institute for Health Development, Kurdistan University of Medical Sciences, Sanandaj, Iran; 4 Department of Epidemiology and Biostatistics, Faculty of Medicine, Kurdistan University of Medical Sciences, Sanandaj, Iran

**Keywords:** Breast Cancer, Meta-analysis, Multiple birth, Twin birth

## Abstract

**Background:**

It has been assumed that perinatal factors such as multiple pregnancies may affect subsequent breast cancer risk in the mother. Considering the inconsistencies in the results of case-control and cohort studies published in the world, this meta-analysis was conducted in order to determine the exact association between multiple pregnancies (twins or more) and the breast cancer incidence.

**Methods:**

This study was performed as a meta-analysis based on PRISMA guidelines by searching the international databases of PubMed (Medline), Scopus, and Web of Science as well as by screening selected articles based on their subject, abstract and full text. The search time was from January 1983 to November 2022. Then the NOS checklist was used to evaluate the quality of the final selected articles. The indicators considered for the meta-analysis included the odds ratio (OR) and the risk ratio (RR) along with the confidence interval reported in the selected primary studies. The desired analyzes were performed with STATA software version 17 to be reported.

**Results:**

In this meta-analysis, 19 studies were finally selected for analysis, which fully met the inclusion criteria. Of these, 11 were case-control studies and 8 were cohort ones. Their sample size was 263,956 women (48,696 with breast cancer and 215,260 healthy) and 1,658,378 (63,328 twin or multiple pregnancies and 1,595,050 singleton pregnancies), respectively. After combining the results of cohort and case-control studies, the effect of multiple pregnancies on the breast cancer incidence was equal to 1.01 (95% CI: 0.89–1.14; I2: 44.88%, P: 0.06) and 0.89 (95% CI: 0.83–0.95; I2: 41.73%, P: 0.07), respectively.

**Conclusion:**

The present meta-analysis results showed, in general, multiple pregnancies were one of the preventive factors of breast cancer.

**Supplementary Information:**

The online version contains supplementary material available at 10.1186/s13690-023-01089-0.

## Introduction

The growing breast cancer prevalence in women is one of the most important problems of humanity in today’s society. In 2020, 2.3 million women were diagnosed with breast cancer and 685,000 died because of its worldwide [1–3]. By the end of 2020, in the last 5 years, 7.8 million women were diagnosed with breast cancer the most common cancer in the world. About 1 in 8 American women (about 13%) develops invasive breast cancer in her lifetime [3]. In 2021, 281,550 new cases of invasive breast cancer and 49,290 new cases of noninvasive breast cancer were estimated in women in the United States [4, 5]. Breast cancer most often begins with cells in the milk-producing ducts (invasive ductal carcinoma). Also, it may begin in the glandular tissue called lobules (invasive lobular carcinoma) or in other cells or tissue within the breast. Results of previous studies showed change of hormonal status, lifestyle and environmental factors that may increase your risk of breast cancer. But it’s not clear why some people who have no risk factors develop cancer, yet other people with risk factors never do. It’s likely that breast cancer is caused by a complex interaction of your genetic makeup and your environment [6, 7]. Many factors are effective in causing breast cancer malignancies, the most important of which are changes in pregnancy patterns and the obesity prevalence [8–10]. In general, these factors include diet, alcohol consumption, body mass index, estrogen consumption, smoking, physical activity, maternal age at the first delivery, menopause, breastfeeding rate, genetic characteristics and family history, race and age at onset of menstruation [11–13]. Epidemiological studies [14] show pregnancy can have different and dual effects on developing tumors as well as increasing the breast cancer risk. On the one hand, after giving birth and in the short term due to cell growth stimulation in the stages of transformation and malignancy, the infection chance increases, and on the other hand, in the long term, we see a decrease in the breast cancer prevalence in mothers because the differentiation of stem cells prone to tumor formation in the breast is intensified following hormonal changes, and as a result, the possibility of malignancy decreases [15, 16]. Furthermore, long-term breastfeeding is associated with a decrease in the breast cancer risk due to the delay in regular ovulation [17]. The results of previous studies have shown there is no clear association between breast cancer and the number of births, age at the time of the last pregnancy, use of birth control pills and hormone replacement therapy in postmenopausal women [18]. In a case-control study, Morabia et al. [19] investigated the breast cancer prevalence and reproductive factors related to it in seven countries (Australia, China, Colombia, Germany, Israel, Philippines and Thailand). The results showed the cancer prevalence was related to early menstruation, late menopause, long duration of pregnancy and more delay in the time of the first delivery. The results of past studies have been completely contradictory. Kim et al. (2012) conducted a meta-analysis to investigate the association between twin births and breast cancer [20]. Although they observed a reduction in the breast cancer risk in the analysis of cohort studies, in general, this association was not statistically significant. This study had some basic limitations. For example, the qualitative evaluation of the selected articles (as the main part of meta-analysis studies) was not properly performed, and subgroup analyzes and meta-regression were not conducted to identify the main heterogeneity sources by identifying confounding variables and controlling their effect. On the other hand, many studies have been published since 2007, which can help in obtaining more accurate information. Therefore, the present meta-analysis aimed to determine the association between multiple births and breast cancer occurrence with the hope that the study results can be effective in health and care programs or interventions for pregnant women and pregnancy outcomes.

## Methods

The present study was a systematic review and meta-analysis based on the structure of Preferred Reporting Items for Systematic Reviews and Meta-Analyses (PRISMA) [21]. The search in the present meta-analysis was performed using the main keywords and their synonyms found by searching in Mesh, Thesauruses, and EMTREE. The desired databases in this study included PubMed (Medline), Scopus, and Web of Science. The present meta-analysis was carried out in order to update the study of Kim, Hye Sook et al., published in 2012 [20]. So, the search time was from January 1983 to November 2022. In order to search, keywords related to twin birth were combined with keywords related to breast cancer and searched in the desired databases. Researchers performed a search of these databases, with hand searching through the reference lists and grey literature. The search protocol, developed based on three main roots of “twin birth”, “multiple birth”, and “breast cancer”. All related components of twin or multiple birth including [(Pregnancies AND Twin), “Twin Pregnancies”, “Twin Pregnancy”, (Pregnancy AND Multiple), “Multiple Pregnancy”, “Multiple Pregnancies”, and (Pregnancies AND Multiple)] and related components of breast cancer including [“Breast Neoplasms”, “Breast Carcinoma”, “Cancer of Breast”, “Breast Malignant Tumor”, “Malignant Tumor of Breast”, “Breast Neoplasm”, “Breast Tumors”, “Breast Cancer”, and “Mammary Cancer”] added to searched queries based on scientific Mesh terms, EMTREE or the key words. The results limited to human subjects and refined for women with breast cancer. Reference Manager bibliographic software was used to manage searched citations. Duplicate entries were searched by considering the title of the published papers, authors, the year of publication, and specifications of the source’s types. In questionable records, the texts were compared. Authors reviewed the primary search results, and after reviewing each article by title and available abstract, some of the articles were eliminated. The evaluation of the papers under consideration was based on the inclusion and exclusion criteria by the researchers, separately (PV, MCH, and YM).

### Inclusion and exclusion criteria

The inclusion criteria were defined based on the PECOT structure [22]. This structure is proper when the objective studies were case-control and cohort. In other word, when the objective of meta-analysis was determined association without any interventions, this structure was used for doing all sections of meta-analysis. The PECOT structure is a helpful approach for summarizing research questions that explore the effect of exposure and is consisted of Population, Exposure (without any intervention), Comparison, Outcomes and Type of studies (22). All case-control and cohort studies which determined the association between the birth of multiple and twin babies and the occurrence of breast cancer met the necessary conditions to enter this study. Other studies with other characteristics and outcomes were excluded from the study. The steps of selecting and screening articles in this meta-analysis were independently performed by two authors (PV and MCH).

### Data extraction

After the screening stage based on the inclusion and exclusion criteria, an information extraction checklist containing information related to studies (including the names of authors, publication year, type of studies, country and number of samples), information related to the desired exposure (singleton, twin or multiple pregnancy), information related to the target population (mothers’ age and body mass index, and the type of population examined in the studies) and information related to the outcome (the desired effect size in the studies along with the 95% confidence interval) was designed, based on which information was extracted from the final articles.

### Quality evaluation of articles

Two of the authors (YM and PV) conducted a qualitative evaluation of the studies on the basis of the Newcastle-Ottawa Quality Assessment Scale (NOS) checklist [23]. This checklist was designed to evaluate the quality of analytical observational studies like case-control and cohort studies. This tool examines each research with eight items in three groups, including how to select study samples, how to compare and analyze study groups, and how to measure and analyze the desired outcome. Each of these items is given a score of one if it is observed in the studies, and the maximum score for each study is 9 points. In case of discrepancies in the score assigned to the published articles, the discussion method and the third researcher were applied to reach an agreement.

### Statistical analysis

In this meta-analysis, two types of case-control and cohort studies were analyzed. The indicators considered for the analysis included the odds ratio (OR) and the risk ratio (RR) along with the confidence interval reported in the selected primary studies. Since these indicators are right-skewed, they should be converted to normal distribution for analysis, and for this reason, the logarithm of these indicators was included in the analysis. The desired model for analysis was random effects or fixed effects (inverse variance). The degree and percentage of heterogeneity in this study were expressed using I square and Cochrane’s Q index [24]. According to the criteria reported by Cochrane [24], 0 to 25% indicates no heterogeneity, 25 to 50% low heterogeneity, 50 to 75% high but acceptable heterogeneity, and 75 to 100% high and unacceptable heterogeneity. In order to evaluate the publication bias, Egger’s test [25] and funnel plot were used. Subgroup analyzes were performed based on type of birth (twin or multiple pregnancies) and different continents.

## Results

**Fig. 1:**
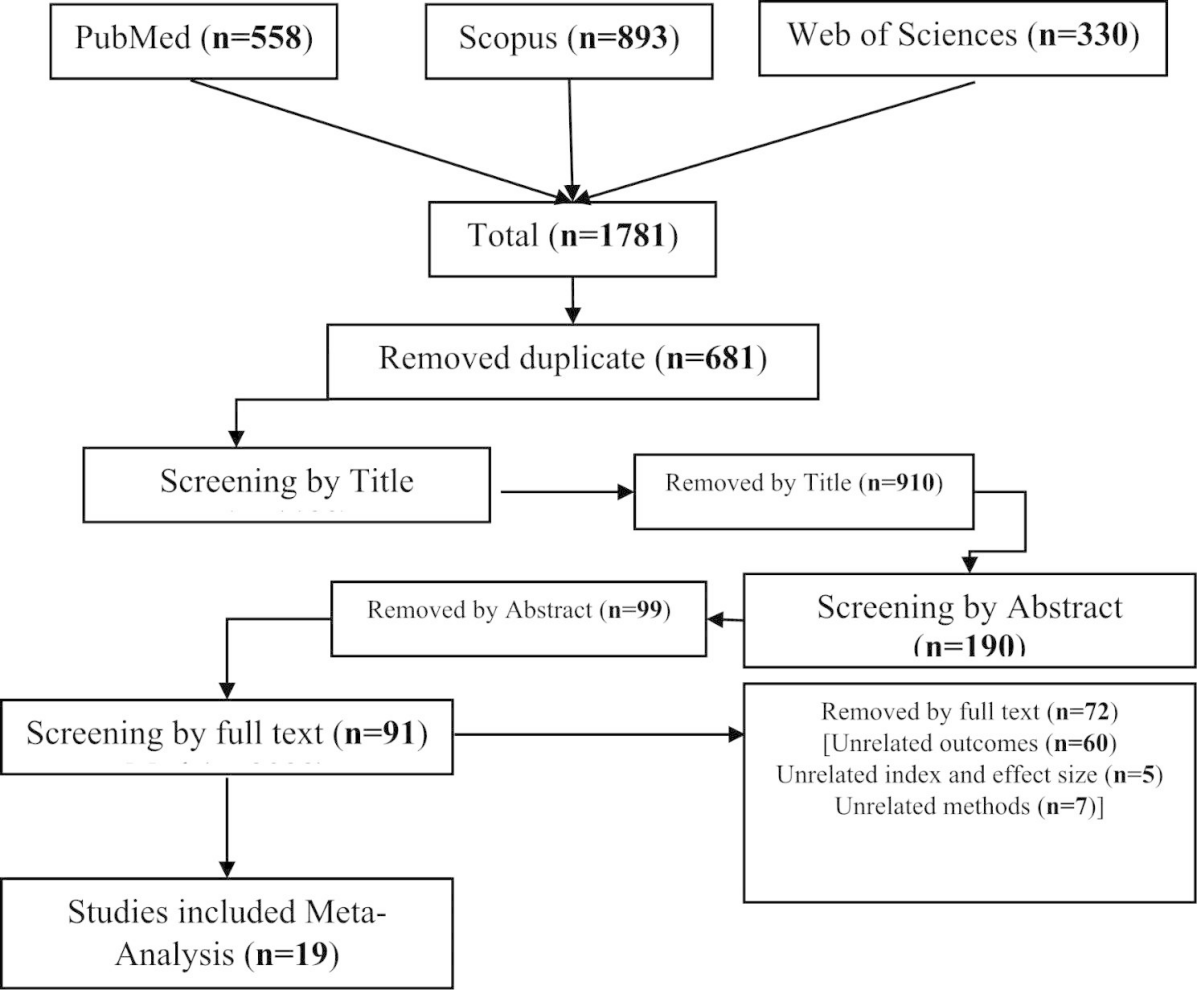
Flow diagram for related article numbers which included in meta-analysis

After completing the search, 558 studies were retrieved in PubMed, 893 in Scopus and 330 in Web of Science. A total of 1781 studies were included in the review, of which 681 were duplicated and in the first step, 1100 studies were entered into the screening stage based on the title. After removing irrelevant studies in this stage, 190 articles were entered into the screening stage based on the abstract. In this step, 99 studies were excluded and in the next step, i.e., screening based on the full text, 91 studies were evaluated (Fig. 1). In this meta-analysis, 19 studies which fully met the inclusion criteria, were finally selected for analysis. Of these, 11 were case-control studies and 8 were cohort ones. Their sample size was 263,956 women (48,696 with breast cancer and 215,260 healthy) and 1,658,378 (63,328 twin or multiple pregnancies and 1,595,050 singleton pregnancies), respectively (Table 1).

**Table 1:** The characteristics of studies (case-control and cohort) on multiple births and maternal breast cancer risk

Authors (Years) (R)	Type of Study (country)	Sample Size Case or exposed/ control or non-exposed	Study population	Mean age Mean BMI	Incidence breast cancer	Effect size (% 95 CI)	NOS Score
I.M. Krul et al., 2014	Cohort (Netherlands)	12,589 (women who had been treated with IVF and completed a risk factor questionnaire) 1688 (with Multiple birth) 10,901 (without Multiple birth)	Dutch women who had been treated with IVF between 1983 and 1995 and completed a risk factor questionnaire between 1997 and 1999	32.9 NR	317 (57 had multiples)	Adjusted hazard ratio (HR) (95% CI): Multiple birth: 1.44 (1.06–1.97) Twins 1.33 (0.95–1.86) Triplets/quadruplets/two multiples 2.19 (1.21–3.94)	8
R. Troisi et al., 2012	Case-control (Norway, Sweden And Denmark)	15,609 women 1419 case (women born in Norway, Sweden or Denmark who were subsequently diagnosed with primary, invasive breast cancer) 14,190 controls (women without breast cancer from the national birth registries matched on birth country, birth year and vital status)	Norwegian, Swedish and Danish Women	32.3 NR	NR	Relative risk (RR) for multiple gestation (95% CI) = 0.81 (0.53–1.23)	7
Lauren E. B. et al., 2018	prospective cohort (USA)	50,983 women 1583 with Twin/triplets 49,400 without Twin/triplets	African American women	41.5 28.3	1583 breast cancer cases among 50,983 cases	Multivariable hazard ratio (MV HR) for Twin/triplet (95% CI): 1.18 (0.84, 1.66)	8
Wyshak et al., 1983	Cohort (USA)	Multiple births 3982 Without Multiple births 3982	Connecticut twin registry and Connecticut tumor registry	59 NR	0.6% on the basis of incidence rates for white females in Connecticut, 1973-77	Relative risk (RR) for Mothers of Dizygotic Twins: RR = 3.2 (1.12, 11.16)	8
Lambe et al., 1996	nested case-control (Swede)	Multiple births/breast cancer 329/19,368 Multiple births/control 2,031/100,459	Swedish Cancer Registry and a nationwide Fertility Registry	NR NR	NR	Breast cancer risk in mothers with multiple live births: OR = 0.88 (0.78, 0.99)	7
Murphy et al., 1997	nested case – control (UK)	Multiple births/breast cancer 64/4,790 Multiple births/control 895/46,751	Swedish cancer registry	NR NR	NR	breast cancer risk in mothers of twins: OR = 0.71 (95% confidence interval 0.55–0.91)	7
Albrektsen et al., 1995	Cohort (Norway)	Multiple births/breast cancer 97/4,782 Singleton births/breast cancer 4,685/4,782	Cancer registry of Norway	NR NR	4,782 cases with breast cancer of 802,269 parous Norwegian women	risk of breast cancer among women ever having had a twin birth: RR = 0.89 (95% CI = 0.73–1.09) risk of breast cancer among women ever having had a multi birth: RR = 1.48 (95% CI = 0.97–2.25)	8
Wohlfahrt et al., 1999	Cohort (Denmark)	Multiple births/breast cancer 168/9,495 Singleton births/breast cancer 9,327/9,495	Danish cancer registry	NR NR	9,495 incident cases of breast cancer of 998,499 women	Risk of breast cancer in Mothers having a multiple birth: Relative risk (RR) = 1.8; 95% confidence interval (CI) = 1.1–2.8).	8
Neale et al., 2004	retrospective cohort study (Swede)	Breast cancer/multiple births 178/5,080 Breast cancer/singleton births 358/10,181	Utah population database Utah cancer registry	62.8 NR	No. of breast cancer cases 178 among 5080 twin mothers	Adjusted RR (95% CI) = 1.02 (0.85–1.22)	8
Neale et al., 2004	Cohort (Swede)	Multiple births/breast cancer 116/6,309 Singleton births/breast cancer 6,193/6,309	Swedish civil birth register Swedish cancer registry	40.1 NR	6309 breast cancer cases (116 of them were twin mothers)	Adjusted RR (95% CI) = 0.91 (0.75–1.09) p-value = 0.27	8
Ji et al., 2007	Cohort (Swede)	Breast cancer/multiple births 1,010/30,409 Breast cancer/singleton births 57,080/1500,000	Swedish family cancer database	NR NR	1010 breast cancer case in 30,409 twin women	RR = 0.85, 95% CI (0.74–0.98)	7
Polednak et al. 1983	Case-control (USA)	Breast cancer 314 Control 314	Cancer registry in New York state	NR NR	NR	OR = 1.33 (0.38, 4.66)	7
Olsen et al. 1988	Case-control (Denmark)	Breast cancer 5,213 Control 20,025	Denmark cancer registry, medical birth registry	NR NR	5213 breast cancer that 141 twins	OR = 1.07 (0.88, 1.30)	7
Innes et al. 2004	Case-control (USA)	Breast cancer 2,522 Control 10,052	New York state birth and tumor registries	37.6 NR	2522 breast cancer 32 twins	OR = 1.43 (0.75, 2.73)	7
Nasca et al. 1992	Case-control (USA)	Breast cancer 2,561 Control 2,616	Two case studies conducted in New York state	NR NR	2561 breast cancers 79 twins	OR = 1.05 (0.77, 1.44)	8
Hsieh et al. 1993	Case-control (USA)	Breast cancer 2,821 Control 8,882	International case–control studies	NR NR	2733 breast cancers 88 twins	OR = 1.21 (0.94, 1.55)	7
Dietz et al. 1995	Case-control (USA)	Breast cancer 5,880 Control 8,217	Tumor registries in Wisconsin, western Massachusetts, Maine and New Hampshire	NR NR	5880 breast cancer 146 twins	OR = 0.94 (0.75, 1.17)	7
La Vecchia et al. 1996	Case-control (Italy)	Breast cancer 2,569 Control 2,588	Large case–control studies conducted in six regions of Italy	55 NR	2569 breast cancers 38 twin birth	OR = 0.74 (0.51, 1.07)	7
Triosi et al., 1998	Case-control (USA)	Breast cancer 1,239 Control 1,166	US cancer registry covering Atlanta, GA; Seattle, WA; New Jersey	NR NR	1239 breast cancers 35 twins	OR = 0.94 (0.58, 1.51)	7

**Fig. 2:**
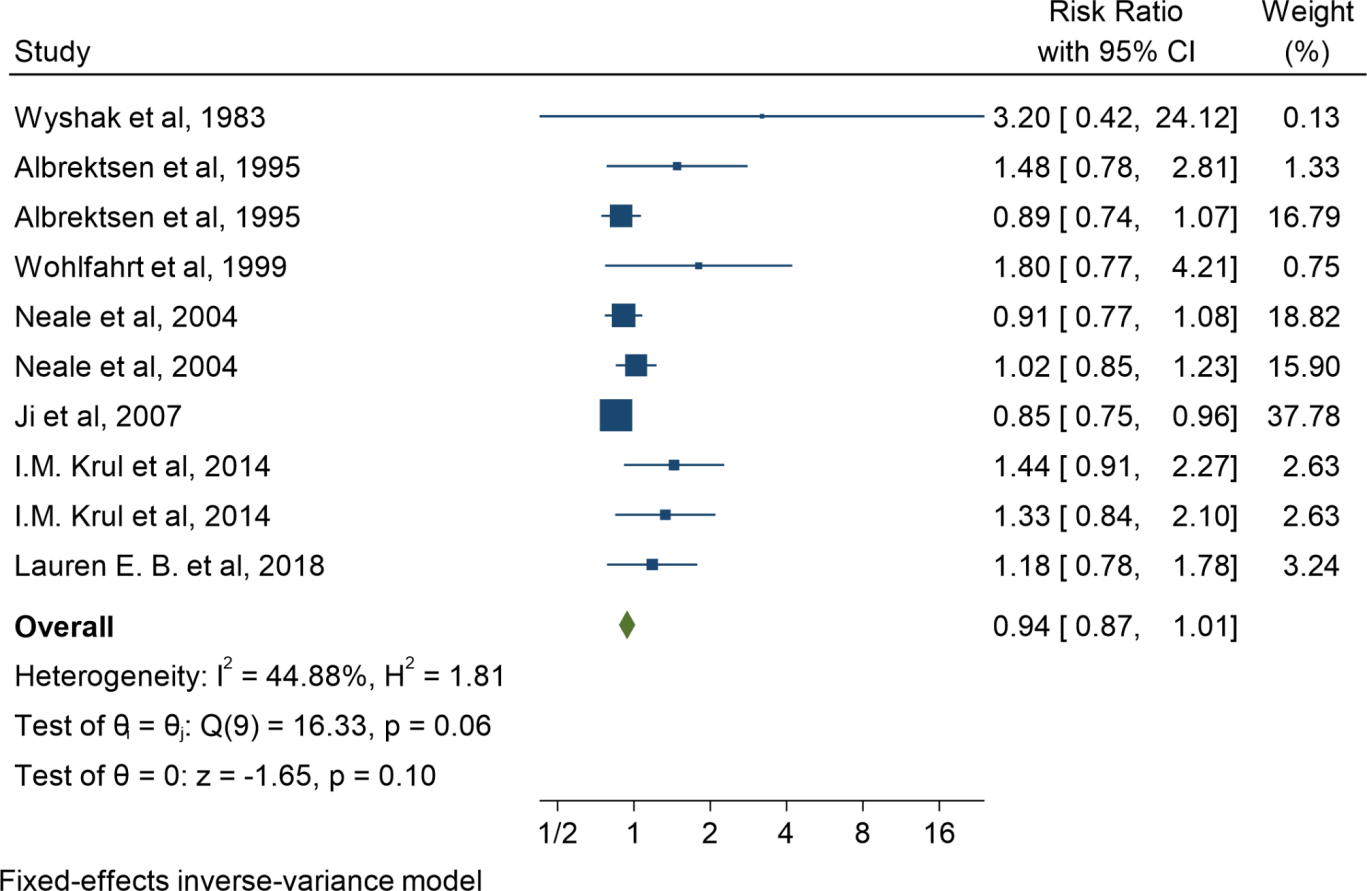
Meta-analysis of the association between multiple pregnancy and maternal risk of breast cancer by combining cohort studies from 1983 to 2022

In the first step of meta-analysis, the results of cohort studies were evaluated and reviewed. From the 8 examined cohort studies, 10 effect sizes including the risk ratio were extracted. The highest and lowest reported associations belonged to the study of Wyshak et al. and Ji et al., respectively. After combining the extracted results, the pooled risk ratio was equal to 1.01 (RR: 1.01; 95% CI: 0.89–1.14; I2: 44.88%, P: 0.06) (Fig. 2).

Subgroup analyzes were performed to determine the association between multiple pregnancies and breast cancer incidence based on the different continents and the type of multiple pregnancies (twins or more) and the results have been reported in Table 2. The results of subgroup analyze after combining cohort studies showed in the American continent, women with multiple pregnancies were 1.27 times more likely to develop breast cancer (RR: 1.27; 95% CI: 0.86–1.88; I2: 62.41%, P: 0.10) while this risk was 1.11 in European women with multiple pregnancies (RR: 1.11; % 95 CI: 1.01–1.34; I2: 77.74%, P: 0.18) (Table 2). Subgroup analysis was also performed based on the type of multiple pregnancies including twins or multiples. The meaning of multiple births was the category of studies which did not specify the exact exposure mode. For example, they did not specify whether the pregnancies were twins or more than twins, like triplets or more. Therefore, they were placed in the multiple birth category. The meta-analysis results showed the association between twin pregnancy and breast cancer incidence was equal to 1.39 (RR: 1.39; 95% CI: 1.14–1.69; I2: 0.00%, P: 0.38) while for multiple pregnancies, this risk was equal to was 0.92 (RR: 0.92; 95% CI: 0.84–1.01; I2: 12.88%, P: 0.16) (Table 2).

**Table 2:** Subgroup analysis of the association between multiple pregnancy and maternal risk of breast cancer by combining cohort/ case-control studies from 1983 to 2022 based on type of birth and continents

Studies	Variables	Pooled Risk Ratio	% 95 Confidence Interval	Heterogeneity assessment between studies		Heterogeneity assessment between subgroup	
				I square	P value	Q test	P value
Cohort	Continents						
	Europe America	0.98 1.27	0.87–1.11 0.86–1.88	45.65% 62.41%	0.18 0.10	0.70	0.40
	Type of Birth						
	Twin Multiple	1.39 0.91	1.14–1.69 0.85–0.99	0.00% 12.88%	0.38 0.16	5.50	0.02
Case-control	Continents						
	Europe America	0.89 1.03	0.79–0.92 0.89–1.18	59.51% 0.00%	0.04 0.79	4.85	0.03
	Type of Birth						
	Twin Multiple	0.90 0.87	0.82–0.99 0.79–0.97	52.22% 0.00%	0.03 0.66	0.22	0.64

**Fig. 3:**
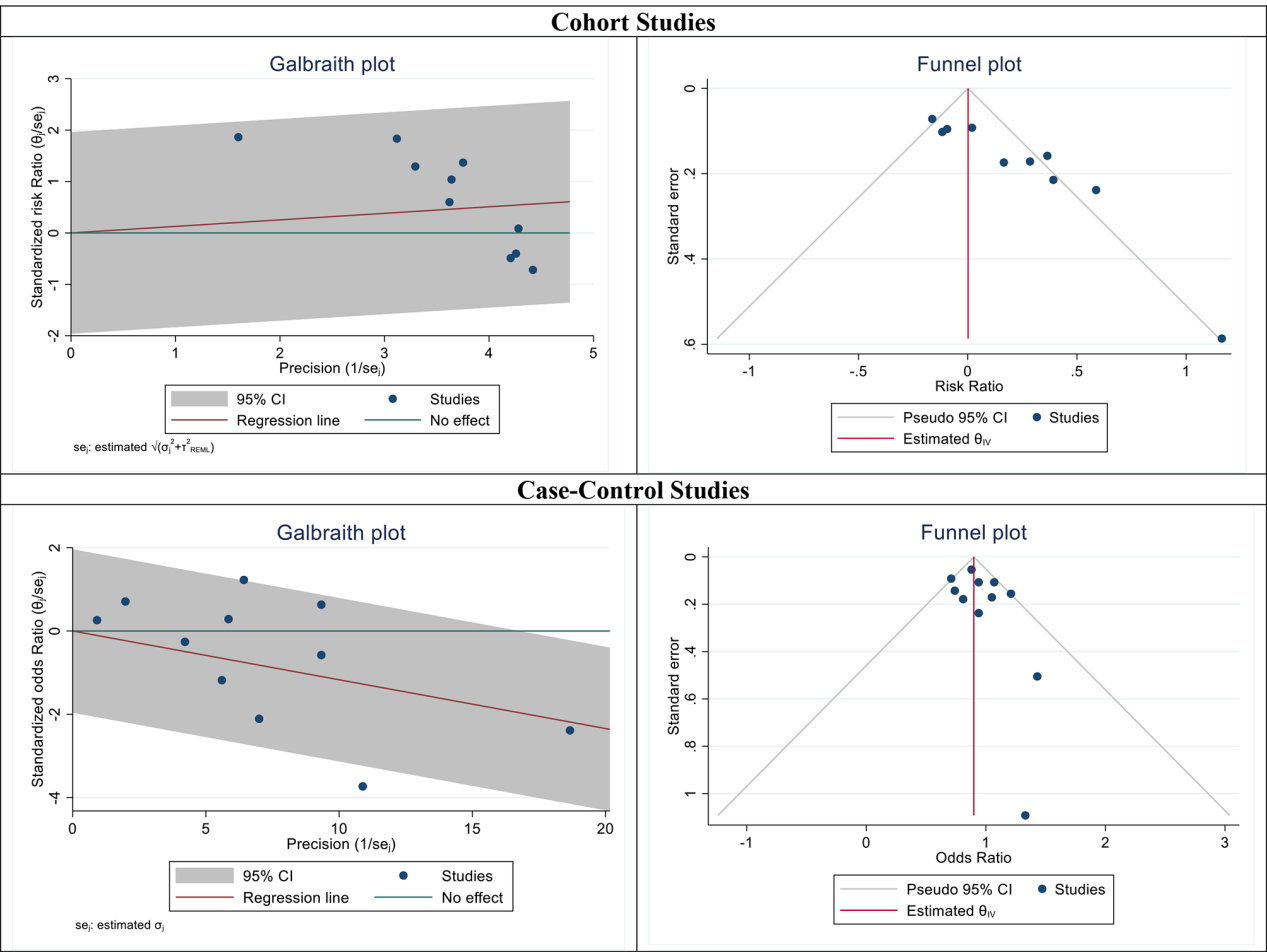
The funnel and Galbraith plots of the association between multiple pregnancy and maternal risk of breast cancer by combining cohort and case-control studies from 1983 to 2022

**Fig. 4:**
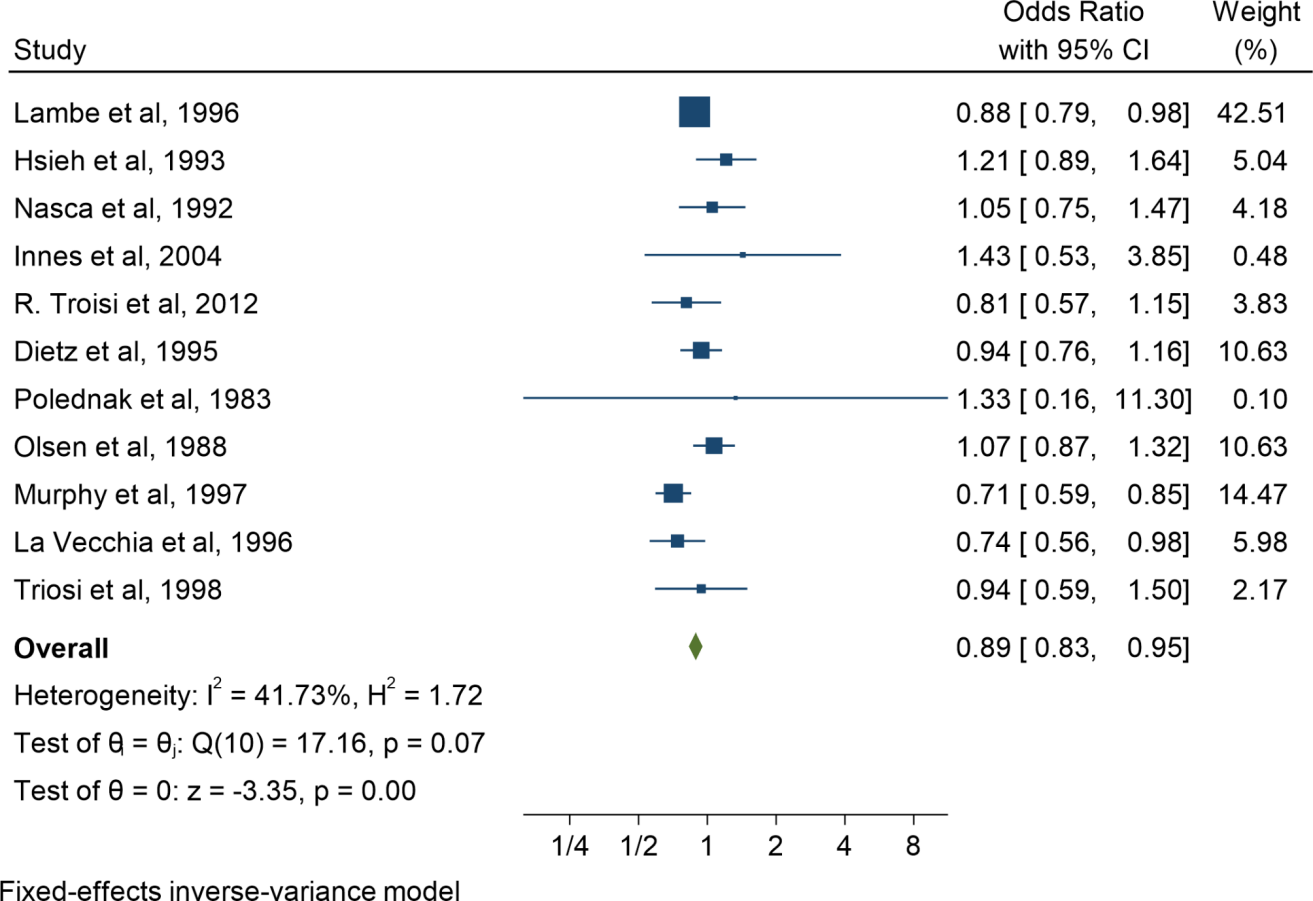
Meta-analysis of the association between multiple pregnancy and maternal risk of breast cancer by combining case-control studies from 1983 to 2022

In the second step of meta-analysis, the results of case-control studies were evaluated. Of the 11 selected studies, the highest and lowest odds ratios were related to the studies of Muphy et al. and Innes et al., respectively. After combining these studies, the pooled OR was equal to 0.89 (OR: 0.89; 95% CI: 0.83–0.95; I2: 41.73%, P: 0.07) (Figs. 3, 4). Subgroup analyzes were performed to determine the association between multiple pregnancies and breast cancer occurrence based on the different continents and the type of multiple pregnancies (twins or more) and the results have been reported in Table 2. The results of subgroup analyze after combining case-control studies showed in the Americas, women with multiple pregnancies were 1.03 times more likely to develop breast cancer (OR: 1.03; 95% CI: 0.89–1.18; I2: 0.00). %, P: 0.79) while in European countries, this risk was lower and equal to 0.89 (OR: 0.89; % 95 CI: 0.79–0.92; I2: 59.51%, P: 0.04) (Table 2). Subgroup analysis based on the type of multiple pregnancies including twins or multiples showed the association between twin pregnancy and the chance of breast cancer was equal to 0.90 (RR: 0.90; 95% CI: 0.82–0.99; I2: 52.22%, P: 0.03) while for multiple pregnancies, this risk was equal to 0.87 (RR: 0.87; % 95 CI: 0.79–0.97; I2: 0.00%, P: 0.66) (Table 2).

## Discussion

The main goal of this meta-analysis was to determine the association between multiple births and the incidence of breast cancer in women. In this meta-analysis, two types of case-control and cohort studies were examined and analyzed. Due to the fact that these two types of studies were different in terms of the nature and method and reporting the effect sizes, we decided to separately report the combination of the results of these two types of studies to determine the association. On the other hand, because breast cancer was not rare in women with multiple births according to the results of previous studies, combining the results of these two types of studies was not correct in terms of methodology and increased the possibility of reporting an unrealistic effect size [26–28]. The effect size is reported in case-control studies as the odds ratio (OR) and in cohort ones as the risk ratio (RR).

The combination of these two indicators is possible only if the desired outcome frequency in the studied population is less than 0.05 or the desired outcome is rare [29, 30]. However, in the present meta-analysis and in the studies which examined the association between multiple births and breast cancer, the prevalence of breast cancer in women with multiple births was higher than 0.05 [31–33]. In the combination of cohort studies, the results showed there was no significant association between multiple births and the occurrence of breast cancer, but the combination of the results of case-control studies showed multiple births (twins or more) significantly reduced the chance of developing breast cancer. This issue can be caused by differences and changes related to pregnancy, which occur in the final pregnancy stages. Although high levels of estrogen, IGF-1 and other cell division stimulators in pregnancy can lead to the stimulation of breast cell proliferation and are a precursor to the initiation and progression towards breast cancer, high levels of HCG and alpha-phytoprotein in pregnancy can have a protective role against breast cancer by increasing apoptosis, inhibiting cell division and enhancing differentiation, and this protective role is often greater in the first pregnancy [34–37].

In addition, according to the results of the study of Janssens, Jaak Ph et al., the HCG hormone has an anti-proliferative role in the laboratory environment on cancer cells [38] and its levels in twin pregnancies are about two times more than that of singleton pregnancies. This can be a justification for the present meta-analysis results [39–42]. In order to confirm these explanations, according to the results of some studies, the levels of AFP produced in the liver and a peptide which inhibits mitogen-activated protein kinase (MAPK or MAP kinase), are higher in multiple pregnancies than in singleton pregnancies. This substance has anti-hormonal effects and can inhibit estrogen-sensitive cells by inactivating the mentioned kinase, neutralizing the effect of estrogen on them and preventing the proliferation of breast cells [41, 43–45].

An increase in the levels of estradiol, testosterone, progesterone, human chorionic gonadotropin and alpha-fetoprotein hormones has been observed and proven in pregnancy, and it seems the increase in human chorionic gonadotropin and fetoprotein progesterone can have a protective effect against breast cancer due to its anti-estrogenic properties effective on the breast tissue, but the association between the higher incidence of breast cancer and the birth of twins or multiples was first established in the 1980s [46]. The physiology of twin and singleton pregnancy differs because higher levels of estradiol and testosterone are observed during twin pregnancy and higher concentrations of follicle-stimulating hormone and sex hormone-binding globulin are seen after twin pregnancy [47]. These changes may affect the incidence of hormone-responsive cancers such as breast, endometrial, and ovarian cancers [48, 49]. Another important point in the current meta-analysis was the existence of a small association between multiple births and breast cancer, which was not statistically significant. In addition, preliminary studies have also shown a significant association in this regard [31, 46, 50], the reason for which can be the higher serum levels of estrogen in multiple pregnancies than in singleton pregnancies. Estrogen stimulates the division of mammary cells and increases hormonal activities such as cytochrome p450 which itself activates metabolic pathways and in this way, it can increase gene mutations and aneuploidy [51–53].

In a similar meta-analysis published by Kim, Hye Sook et al., analyzing 17 articles published from 1983 to 2007, different results were obtained [20]. After combining the results, Kim, Hye Sook et al. showed twin birth was not associated with a reduced incidence of breast cancer. However, subgroup analyzes for cohort studies in this research showed the breast cancer risk tended to decrease in women with a history of multiple births. In the mentioned meta-analysis, the association between multiple births, twin births and breast cancer was not separately stated, but in our meta-analysis, in addition to the association between multiple births and breast cancer, the association between twin births and breast cancer was separately investigated in subgroup analyses. Also, another advantage of the current study was to perform subgroup analyzes to separately determine the association between multiple births, twin births and breast cancer in different continents. Also, different guidelines in the field of breast cancer need to update the information, and the present meta-analysis results can be suitable for updating the information of these guidelines.

One of the current meta-analysis limitations was the lack of subgroup analyzes based on important variables such as receiving treatment, the type of treatment, time and method of cancer diagnosis, body mass index and age which were not examined due to non-reporting or incomplete reporting in the initial studies.

## Conclusion

The present meta-analysis results showed, in general, multiple pregnancies were one of the preventive factors of breast cancer, but information on twin pregnancies was conflicting. Therefore, it is necessary to conduct more cohort and case-control studies with appropriate sample sizes, taking into account important and effective factors such as genetics, age, body mass index, receiving treatment and type of treatment.

## Electronic supplementary material

Below is the link to the electronic supplementary material.

Supplementary Material 1

## Data Availability

and other materials. Data and materials are available within the supplementary materials, and further information can be made available by request to the corresponding author.

## Abbreviations

CI: Confidence Interval
OR: Odds Ratio
RR: Risk Ratio/Relative Risk
I^2^: I Square
NOS: Newcastle Ottawa Scale
PRISMA: Preferred Reporting Items for Systematic Reviews and Meta-Analyses
