# Supplementary material for: The association between the multiple birth and breast cancer incidence: an update of a systematic review and meta-analysis from 1983 to 2022
Source: Arch Public Health. 2023 Apr 28;81:76. doi: 10.1186/s13690-023-01089-0 (PMC10142199; doi:10.1186/s13690-023-01089-0)
Supplement: Supplementary file 1 — Supplementary Material 1 [file 13690_2023_1089_MOESM1_ESM.docx]

**Statement**

**What is known:**

Furthermore, long-term breastfeeding is associated with a decrease in the breast cancer risk due to the delay in regular ovulation. The results of previous studies have shown there is no clear association between breast cancer and the number of births, age at the time of the last pregnancy, use of birth control pills and hormone replacement therapy in postmenopausal women. The results of past studies have been completely contradictory. On the other hand, many studies have been published since 2007, which can help in obtaining more accurate information. Therefore, the present meta-analysis aimed to determine the association between multiple births and breast cancer occurrence with the hope that the study results can be effective in health and care programs or interventions for pregnant women and pregnancy outcomes.

**What does this study add:** The present meta-analysis results showed, in general, multiple pregnancies were one of the preventive factors of breast cancer around the world. The results of subgroup analyze after combining cohort studies showed in the American continent, women with multiple pregnancies were 1.27 times more likely to develop breast cancer while this risk was 1.11 in European women with multiple pregnancies. Also, results showed the association between twin pregnancy and breast cancer incidence was equal to 1.39 while for multiple pregnancies, this risk was equal to was 0.92.

**What are implications for clinical practice, public health and / or research:** The present meta-analysis results showed, in general, multiple pregnancies were one of the preventive factors of breast cancer, but information on twin pregnancies was conflicting. Therefore, it is necessary to conduct more cohort and case-control studies with appropriate sample sizes, taking into account important and effective factors such as genetics, age, body mass index, receiving treatment and type of treatment. Broadening the scope of research might yield additional important information on the etiology and of ways to prevent breast cancer. Findings from this meta-analysis suggest that the entire life course of a woman's life, including her prenatal experience, might be relevant for her future breast-cancer risk. Research efforts might focus on ascertaining the most important windows of susceptibility to identify new opportunities for prevention. Future studies need to confirm the importance of early-life exposures for the risk of breast cancer and identify underlying mechanisms.
